# Label-free proteomic analysis of Duchenne and Becker muscular dystrophy showed decreased sarcomere proteins and increased ubiquitination-related proteins

**DOI:** 10.1038/s41598-025-87995-5

**Published:** 2025-01-26

**Authors:** Juliana Cristina Tobar da Silva, Mariângela Rangel Alves Nogueira, Yara Martins da Silva, Fábio César Sousa Nogueira, Nathalie Henriques Silva Canedo, Katia Carneiro, Denise de Abreu Pereira

**Affiliations:** 1 Graduate Course in Medicine (Pathological Anatomy), Federal University of Rio de Janeiro, Rio de Janeiro, Brazil; 2 Proteomics Unit, Department of Biochemistry, Institute of Chemistry, Rio de Janeiro, Brazil; 3 Proteomics Laboratory (LabProt), LADETEC, Institute of Chemistry, Federal University of Rio de Janeiro, Rio de Janeiro, Brazil; 4 Center for Precision Medicine, Carlos Chagas Filho Institute of Biophysics, Federal University of Rio de Janeiro, Rio de Janeiro, Brazil; 5 Cellular and Molecular Oncobiology Program, Research and Innovation Coordination, National Cancer Institute- INCA/RJ, Rio de Janeiro, Brazil; 6 Laboratory of Cellular Proliferation and Differentiation, Institute of Biomedical Sciences, Federal University of Rio de Janeiro, Rio de Janeiro, Brazil

**Keywords:** Proteomics, Musculoskeletal system

## Abstract

Muscular dystrophies (MD) are a group of hereditary diseases marked by progressive muscle loss, leading to weakness and degeneration of skeletal muscles. These conditions often result from structural defects in the Dystrophin–Glycoprotein Complex (DGC), as seen in Duchenne Muscular Dystrophy (DMD) and Becker Muscular Dystrophy (BMD). Since MDs currently have no cure, research has focused on identifying potential therapeutic targets to improve patients’ quality of life. In this study, skeletal muscle tissue samples from DMD and BMD patients, as well as non-dystrophic controls, were analyzed using label-free mass spectrometry (MS/MS) to characterize the proteomic profile of these conditions and identify biomarkers for differential diagnosis. In-silico analysis revealed that dystrophic muscle tissues are linked to biological processes related to cellular energy metabolism, including oxidation of organic compounds, energy production, and cellular respiration. Enrichment of functions associated with cell structure and RNA binding was also observed, including cytoskeletal protein binding and RNA binding. The human phenotypes most related to the proteomic signature were abnormal circulating metabolites, muscle physiology, and weakness. Quantitative analysis identified significant changes in proteins associated with sarcomere organization and protein ubiquitination, such as myomesin, myozenin, and E3 ubiquitin-protein ligase rififylin, suggesting these as potential therapeutic targets.

**Supplementary Information:**

The online version contains supplementary material available at 10.1038/s41598-025-87995-5.

## Introduction

Skeletal muscles (SM) constitute a significant portion of the human muscular mass, comprising about 40% of body weight. SM accounts for approximately 50–75% of the body’s proteins and consists of 75% of water, 20% of proteins, including inorganic salts, minerals, fats, and carbohydrates, which together represent about 5% of the constituents of SMs^1^. The histological architecture of SM is characterized by the presence of cylindrical, multinucleated cells, composed by myofibrils, which are formed by repeated contractile units responsible for the muscle contraction process called sarcomeres^2^. The Dystrophin–Glycoprotein Complex (DGC) is the major structural element present in the muscle fibres and is responsible for maintaining the stability and integrity of the fibres through a network that couples the extracellular matrix components to the cytoskeleton^3^. This complex consists of Dystrophin, which in turn, binds to transmembrane sub-complexes composed by dystroglycans, sarcoglycans, and sarcospan, as well as cytoplasmic proteins such as syntrophin, dystrobrevin, and neuronal nitric oxide synthase (nNOS)^4,5^. Muscular Dystrophies (MD) represent a heterogeneous group of hereditary diseases characterized by progressive loss of muscle tissue leading to weakness and degeneration of skeletal muscles^6^. Most muscular dystrophies occur due to structural alterations in DGC proteins, such as in Duchenne Muscular Dystrophy (DMD) and Becker Muscular Dystrophy (BMD).

DMD is a lethal muscular disease with a prevalence of 4.8 individuals/100,000^7^. Clinically, the first signs of DMD can be observed before the age of five. The main signs include frequent falls, difficulty in climbing stairs, running, getting up from the floor, scoliosis accompanied by hyperlordosis, and calf hypertrophy^8^. DMD is characterized by symmetrical and progressive loss of muscle mass, starting at the pelvic girdle (hips and legs), later affecting the upper limbs. Fiber degeneration, and subsequent fibrosis, result in gait disturbances in early childhood, followed by loss of ambulation and cardiopulmonary complications^9^. The severity of the disease progresses with age, and patients become wheelchair-bound, with some cases requiring mechanical ventilation^10^.

Patients with DMD present a variable phenotype^11^, which is characterized by progressive and irreversible degeneration of skeletal muscle^12^, ranging from gait disturbance in early childhood to nearly a small weakness in adulthood^13^. Typically, DMD onset is juvenile accompanied by muscular weakness, calf hypertrophy, with gradual progression that can lead to loss of motor function and cardiomyopathy^14^.

BMD is a milder and less lethal form of the disease with a later age of onset, also presenting an indolent clinical progression. BMD presents a global prevalence estimated at 1.6/100,000^7^. In addition to differences in incidence and clinical manifestations, these two types of dystrophies are genetically distinct. While DMD often presents frame-shift mutations, abolishing dystrophin expression or generating extremely low amounts of dystrophin protein, BMD presents in frame deletions mutations that lead to protein truncations, thus generating an aberrant dystrophin protein with altered functions^5,15^. These alterations typically lead to a pattern of reduced anti-Dystrophin immunostaining in BMD and absence in DMD. Interestingly, both diseases are caused by different types of mutations in the gene encoding the Dystrophin protein^16,17^. The absence of dystrophin destabilizes the DGC, leading to increased fragility and permeability of the plasma membrane, resulting in dysregulation of calcium levels and increased oxidative damage^18,19^. Necrosis is closely associated with increased inflammation and oxidative stress, which in turn leads to regenerative myogenesis. Furthermore, the progressive increase in fibrosis caused by myonecrosis and inflammation impairs myogenesis and muscle regeneration, resulting in severe degeneration of muscle tissues. As patients age, the regenerative capacity of muscle decreases, and associated connective tissue gradually replaces muscle fibers^20^.

Like all degenerative diseases, MDs have no cure, and for this reason, clinical and experimental studies have focused on characterizing putative therapeutic targets to increase the time and quality of life for patients^21^. Although advances in genetic sequencing have successfully mapped and identified genetic mutations related to MDs, especially for DMD, the genetic and clinical heterogeneity of this group of diseases makes it indispensable to better characterize the underlying pathophysiological mechanisms of the degenerative process. For instance, genetic sequencing studies for Limb-Girdle Muscular Dystrophy (LGMD) have shown that BMD is often underdiagnosed, with patients being diagnosed as limb-girdle dystrophy^22,23^.

We have observed a consistent increase in proteomic studies related to muscle diseases. However, many of these studies are conducted using animal genetic models, human plasma, urine or even cell culture^24,25^. Although different proteomic studies have successfully employed label-free mass spectrometry technique for investigating muscular dystrophies in experimental models^26,27^, this powerful approach has proven to be limited for application in humans due to evident constraints on sample collections from human tissue biopsies. Therefore, the search for more accurate diagnostic tools, which can highlight unique characteristics of each type of dystrophy, is extremely relevant today. In this context, proteomic analysis stands out as an important strategy for the identification and characterization of protein biomarkers that can significantly contribute both to a more detailed understanding of the physiology and homeostasis of healthy muscle as well, and to a more accurate histopathological diagnosis of MDs. It is important to note that although biopsy is an invasive technique, it is still widely used and economically viable for the histopathological study of MDs using histochemical and immunohistochemical markers. These features may facilitate multi-omics approaches to better characterize the main clinical and physiological aspects of dystrophies.

In this work, skeletal muscle tissue samples from DMD and BMD patients obtained by biopsy, as well as from non-dystrophic patients, were used. The tissues were analysed using a label-free mass spectrometry MS/MS proteomic methodology to characterize the proteomic profile of DMD and BMD both qualitatively and quantitatively and search for putative useful biomarkers for the differential diagnosis of dystrophies. Our in-silico analysis showed that the molecular signature of dystrophic muscle tissue is related to biological processes associated with cellular energy metabolism, such as energy derivation by oxidation of organic compounds, generation of precursor metabolites and energy, and cellular respiration. We also observed an enrichment of molecular functions related to cell structure and RNA binding, such as structural molecule activity, cytoskeletal protein binding, and RNA binding. In fact, the human phenotypes most related to the proteomic signature of DMD were associated with abnormal circulating metabolite concentration, abnormal muscle physiology and muscle weakness. Quantitative analysis showed that Becker and Duchenne muscular dystrophy significantly alter the abundance of proteins related to sarcomere organization and protein ubiquitination as myomezin, myozenin and E3 ubiquitin-protein ligase rififylin suggesting them as putative therapeutic targets.

## Methodology

All samples in this study were obtained in the Neuropathology sector of the Clementino Fraga Filho Hospital (HUCFF)at Federal University of Rio de Janeiro, between 2011 and 2021. The protocol (129/2023) was approved by the Ethics Committee of the HUCFF (approval number 6,528,766). Although researchers provided informed consent to all patients, our sample exhibited a high mortality rate, and some patients had outdated contact information, rendering them unreachable. In accordance with the Ethics Committee guidelines and Brazilian legislation, the Ethics Committee of the HUCFF authorized the utilization of stored human biological samples. We ensure the privacy and confidentiality of the research regarding the personal data ensuring that the patients were not identified in the research. All experiments followed the guidelines and regulations in accordance with the Declaration ofHelsinki.

We used 4 samples of human muscle tissue obtained by biopsies from patients diagnosed with DMD and 3 samples from patients diagnosed with BMD, patients’ age ranges from 5 to 11 years. For the Control group, 3 samples of muscles were used from non-dystrophic patients. All the biopsies were collected from biceps or quadriceps femoral muscles. Biopsy samples were removed from liquid nitrogen where they were stored and sectioned in a cryostat into 20 slices measuring 20 μm each. The sections were immediately placed in Eppendorf microtubes and kept in liquid nitrogen to be further processed for mass spectrometry analysis.

### Extraction of skeletal muscle proteins

To remove red blood cells, two washes with phosphate buffered saline (PBS) were performed, followed by centrifugation for 5 min at 1,500 rpm at 4 °C, with the supernatant discarded after each wash. The pellets immersed in liquid nitrogen were macerated with a plastic pestle, and then the lysis buffer, adapted from Kolbel et al.^28^(50 mM Tris-HCl pH 7.8, 150 mM NaCl, 1% SDS, and Halt Protease Inhibitor Cocktail—Thermo Scientific), was added. Subsequently, the samples were sonicated for 5 s on ice, three times, with a 1-minute rest between each sonication. After vortex homogenization for 1 min, with 5-minute intervals, three times, the samples were centrifuged at 20,000 g for 20 min at 4 °C. The resulting supernatant was collected, and proteins were quantified using the BCA Protein Assay colorimetric kit (Thermo Scientific, Ref. 23228) exactly as recommended by the fabricant using a Spectramax 100 reader at a wavelength of 562 nm.

### Protein hydrolysis

The protein extracts were hydrolysed according to the “filter-aided sample purification” (FASP) protocol by Wiśniewski et al.^29^. In brief, 50 µg of total proteins from tissue lysates were incubated for 30 min at 60 °C in the presence of 10 mM Dithiothreitol (DTT), after this the samples were centrifuged at 3,000 rpm for 2 min at 20 °C. Following that, the samples were subjected to two washes in 8 M Urea in 0.1 M Tris/HCl pH 8.5 by centrifugations at 14,000 g for 40 min. Thereafter, the samples were incubated with 50 mM Iodoacetamide for 20 min. After that, the samples were centrifuged for 30 min at 14,000 g, and the supernatant was discarded. The pellet was submitted to two washes with 8 M Urea in 0.1 M Tris/HCl pH 8.0 followed by centrifugation for 30 min at 14,000 g. The pellets were then subjected to enzymatic digestion with Lys-C Mass Spec (Promega) in 8 M Urea and 0.1 M Tris/HCl pH 8.0 at an enzyme/protein ratio of 1:50. The samples were homogenized for 1 min on a thermo-shaker (Nova Instrument) at 600 rpm and incubated for 20 h. After incubation, the filters were transferred to new microtubes, where the second enzymatic digestion step was performed with Trypsin (Promega) in 50mM Ammonium Bicarbonate, at an enzyme/protein ratio of 1:100. The samples were homogenized for 1 min on the thermo-shaker (Nova Instruments) at 600 rpm and incubated at 37 °C for 4 h, followed by centrifugation for 40 min at 14,000 g. Next, 50 µL de NaCl 0,5 M were added and after centrifugation at 14.000 g for 20 min the filtered peptides were collected and acidified with TFA at 10%.

### Peptides purification

The peptides were concentrated, desalted, and eluted using 100 µL C18 tips (Thermo Scientific Ref.87784) exactly as recommended by the fabricant. In short, 100 µL of 50% Acetonitrile (ACN) was aspirated and discarded, this process was repeated twice. Next, 100 µL of 0.1% Trifluoroacetic Acid (TFA) were aspirated and discarded, twice. After this step, 100 µL of the protein extract was aspirated, and the solution was homogenized with the pipette for better sample elution. Next for the rinse step, 100µL of 0.1% TFA solution in 5% ACN was used. Then the peptides were eluted with 100 µL of 0.1% Formic Acid in 75% ACN and the samples were completely dehydrated using the Speed vac (Thermo Scientific) and sent to LADETEC at Instituto de Química, da Universidade Federal do Rio de Janeiro for analysis by NANOLC-MS/MS.

### Mass spectrometry

The mass spectrometry analysis was done using an Easy1000 nanoLC system (Thermo Fisher) coupled to a Quadrupole Orbitrap mass spectrometer (Q Exactive Plus, Thermo Scientific). The dried peptides were solubilized in 0.1% formic acid and a volume of 4 µL was applied to a Trap column with an internal diameter of 200 μm and a length of 2 cm packed with Reprosil-Pur C18 resin (Dr. Maisch), with pores of 200 Å and particle size of 5 μm (packaged in the laboratory). The analytical column was packed with Reprosil-Gold C18 resin (Dr. Maisch), with 300 Å pores and 3 μm particle size (packaged in the laboratory) with 75 μm in diameter and 25 cm in length. Peptide elution was performed using a gradient from 98% solvent A (5% ACN and 0.1% formic acid) to 20% solvent B (95% ACN and 0.1 formic acid) for 85 min, 20–40% solvent B in 22 min and 40–95% solvent B in 5 min. After that, the column was re-equilibrated with solvent A. The Orbitrap mass spectrometer was controlled by the Xcalibur 2.2 software, which was programmed to operate in the data dependent acquisition (DDA) mode. The MS1 spectrum was acquired with a resolution of 70,000 at 200 m/z (mass/charge). MS1 spectrum reading was performed using 106 ions (AGC) and 50 ms Maximum IT. The reading spectrum comprised ions with 375 to 2000 m/z. The 20 most intense ions were fragmented and then subjected to MS2 acquisition, using collision induced dissociation (HCD) and 200–2000 m/z range. MS2 resolution was 17,500 to 200 m/z, AGC of 105 ions, Maximum IT of 100 ms. Ion isolation window was 1.2 m/z, normalized collision energy (NCE) was 30, dynamic exclusion time was 60s. Peptides with undetermined charges and + 1 were rejected. The samples (3 Controls, 4 DMD and 3 BMD) were injectedin technical duplicates for each biological sample.

Database searches and identification of peptides and proteins were conducted using Proteome Discoverer v2.5.0.400. Searches were performed against a concatenated Homo sapiens (human) database downloaded from UniProt (The UniProt Consortium, 2022) database (reference proteome, 82,485 entries) and contaminants list, utilizing the Sequest HT algorithm. The precursor mass tolerance used was 10 ppm, fragment mass tolerance of 0.1 Da, fixed modification of carbamidomethyl (Cys) and variable modification of N-terminal acetylation, Met-loss (Met), Met-loss + acetyl (Met) and oxidation (Met). Full tryptic cleavage specificity where two missed cleavage sites were allowed. False discovery rates (FDR) were obtained using Percolator and the proteins considered have medium and high confidence.

### Experimental design and statistical analysis

The data obtained from mass spectrometry were filtered using the Interactive Venn software (https://www.interactivenn.net/) to identify common proteins among the samples of each experimental group. The proteins common to the experimental groups C1, C2, and C3 formed the Control Group (CG), the proteins common to the experimental groups D1, D2, D3, and D4 made up the Duchenne Muscular Dystrophy (DMD) group, and the proteins common to the experimental groups B1, B2, and B3 were selected as the Becker Muscular Dystrophy (BMD) group. Then using these filtered proteins, the abundance of each biological sample (obtained by the average of the technical duplicate) was considered for the statistical analysis used to identify the increase and decrease in protein abundances. Statistical analysis was carried out in Perseus v 1.6.2.2 and R. Protein area values were converted to log2 scale and normalized by subtracting the median of the sample distribution. Proteins with < 50% valid values in each group (Control, DMD and BMD) were removed from the analysis. The remaining proteins were subjected to missing value imputation using the default parameters (width 0.3, down shift 1.8) of the Perseus Imputation tool.

### In silico analysis

All the proteins identified in each experimental group were analysed in the STRING platform (https://string-db.org/) to evaluate the gene ontology distribution in biological processes and in molecular functions. The association with human phenotype was analysed based on the Monarch Initiative platform (https://monarchinitiative.org). In each of these gene ontology annotations, the most enriched biological processes, molecular functions, and human phenotypes were plotted according to the False Discovery Rate (FDR) value. The most enriched pathways in each experimental group were analysed in the reactome platform (https://reactome.org/). The InteractVenn platform was used to show the distribution of the identified proteins between the groups (https://www.interactivenn.net/).

Heatmaps were done with Morpheus (https://software.broadinstitute.org/morpheus/) to show statistically significant proteins with abundance increased or decreased.

## Results

### Characterization of the molecular signature of muscle tissues

Although the DGC has been well characterized as the major progressive structural alterations responsible for the BMD and DMD the search for new proteins is still necessary to better understand these structural changes allowing the search for mechanisms that lead to reversing or even slowing down disease progression.

In our proteomic analysis a total of 1,649 proteins were identified for all the three experimental groups including control, Becker and Duchene dystrophies (Supplementary Table 1). A total of 1,126 proteins were identified in control 1 (C1), 1,266 proteins were identified in control 2 (C2) and 1,413 proteins were identified in control 3 (C3). Among these, 960 proteins were common to all 3 controls and will be referred to as control group (CG) (supplementary Fig. 1 and Supplementary Table 2). In Becker Muscular Dystrophy 1 (B1) 1,278 proteins were identified, in BMD 2 (B2) 1,303 proteins were identified and in BMD 3 (B3) 1,055 proteins were identified. Among these, 867 proteins were common to all the 3 Becker patient samples and will be referred to as BMD group (BG) (supplemental Fig. 1 and Supplemental Table 2). In Duchenne Muscular Dystrophy 1 (D1) 1,372 proteins were identified, in DMD 2 (D2) 995 proteins were identified, in DMD 3 (D3) 1,278 proteins were identified and in DMD 4 (D4) 1,057 proteins were identified. Among these, 801 proteins were common to all the 4 DMD patients and will be referred to as DMD group (DG) (supplemental Fig. 1 and Supplemental Table 2).

#### The most enriched biological processes were related to cellular energy metabolism

**Fig. 1:**
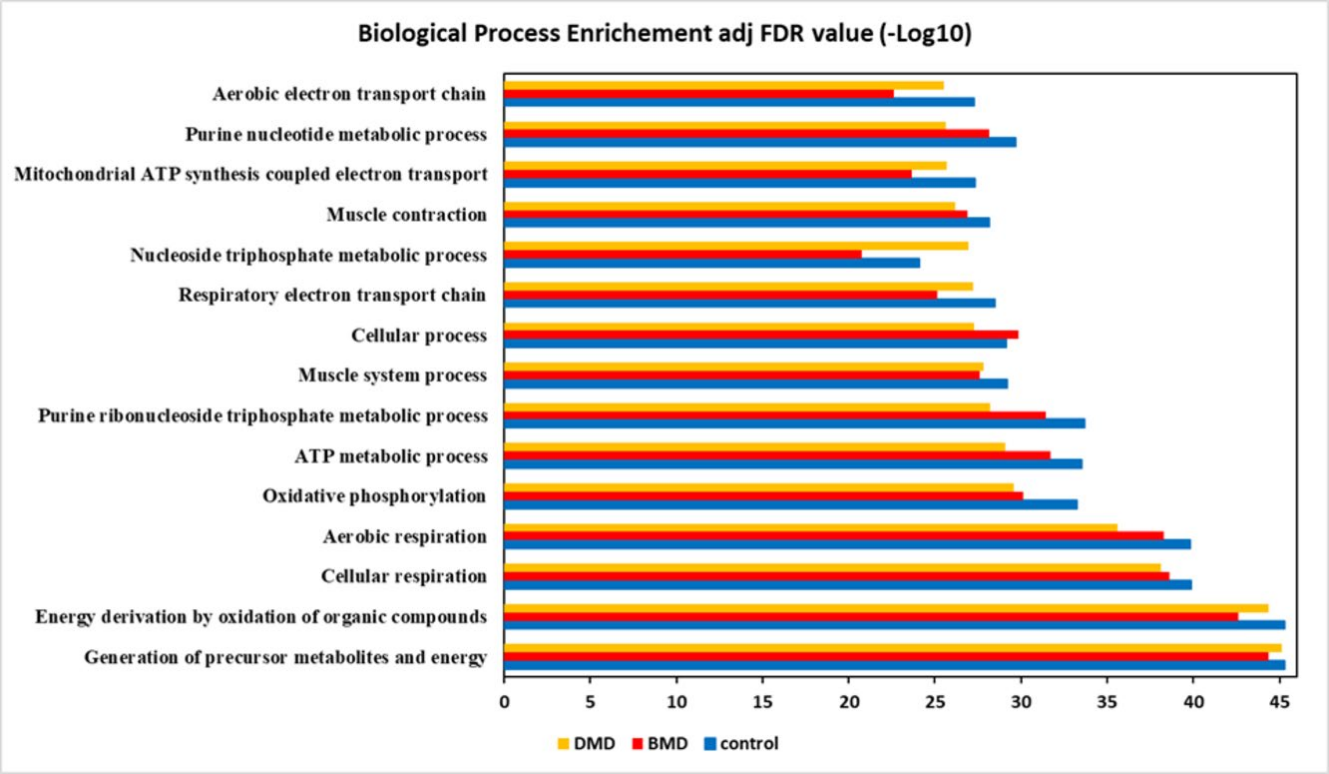
GO String classification showing the biological process enrichment for the top fifteen biological processes based on the adjusted (adj) FDR value.

To characterize the molecular signature of the muscle samples all the proteins identified in each experimental group were examined according to the STRING GO classification. Analysing the biological process, we observed that the three most enriched processes, based on the FDR value, were associated with cellular energy metabolism, such as generation of precursor metabolites and energy (GO:0006091), energy derivation by oxidation of organic compounds (GO:0015980) and cellular respiration (GO:0045333) (Fig. 1; Supplemental Table 3). The biological process of Energy derivation by oxidation of organic compounds (GO:0015980) is represented by 96 proteins identified in CG, 87 proteins identified in DMD, and 88 proteins identified inBMD (Supplemental Table 4). The biological process Generation of precursor metabolites and energy (GO:0006091) is represented by 115 proteins in CG, 105 proteins in DMD, and 108 proteins in BMD (Supplemental Table 4). The biological process Cellular respiration (GO:0045333) is represented by 79 proteins in CG, 71 proteins in DMD, and 74 proteins in BMD (Supplemental Table 4).

#### The most enriched molecular functions were related to structural molecule activity, cytoskeletal protein binding, and RNA binding

**Fig. 2:**
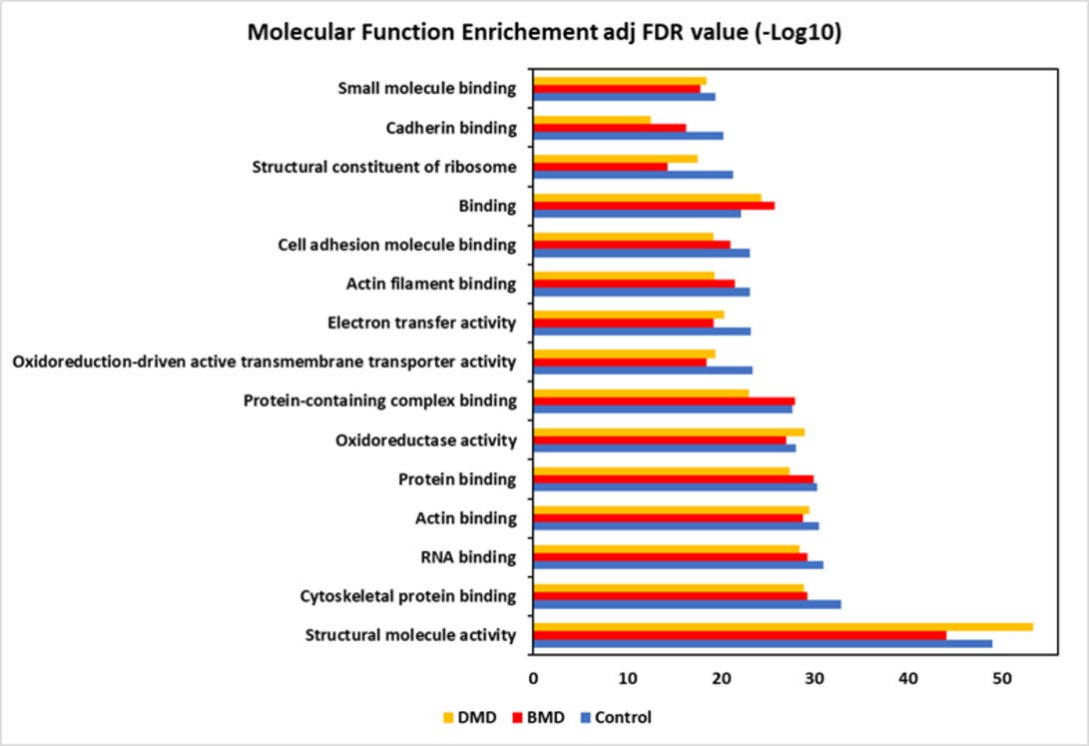
GO String classification showing the molecular function enrichment for the top fifteen molecular functions based on the adjusted (adj) FDR value.

Next, we used the STRING GO classification system to assess the enrichment of molecular functions for all the proteins identified in each group. We observed that the most enriched molecular functions were related to cell structure and oxidative stress, such as structural molecule activity (GO:0005198), cytoskeletal protein binding (GO:0008092), and RNA binding (GO: GO:0003723) (Fig. 2; Supplemental Table 5). We identified 158 proteins in the molecular function related to structural molecule activity in CG, 149 proteins in DMD, and 142 proteins inBMD (Supplemental Table 6). For the molecular function of cytoskeletal protein binding, 151 proteins were found in GC, 128 proteins in DMD, and 135 proteins in BMD (Supplemental Table 6). For the molecular function of RNA binding, 197 proteins were found in CG, 169 in DMD, and 180 proteins inBMD (Supplemental Table 6).

#### The human phenotypes most enriched were related to abnormal circulating metabolite concentration, muscle weakness, and abnormal muscle physiology

To further investigate the relationship of the identified proteins in the experimental groups with diseases, we used the Monarch initiative platform, a challenging way to integrate genetic data, phenotype and diseases.

**Fig. 3:**
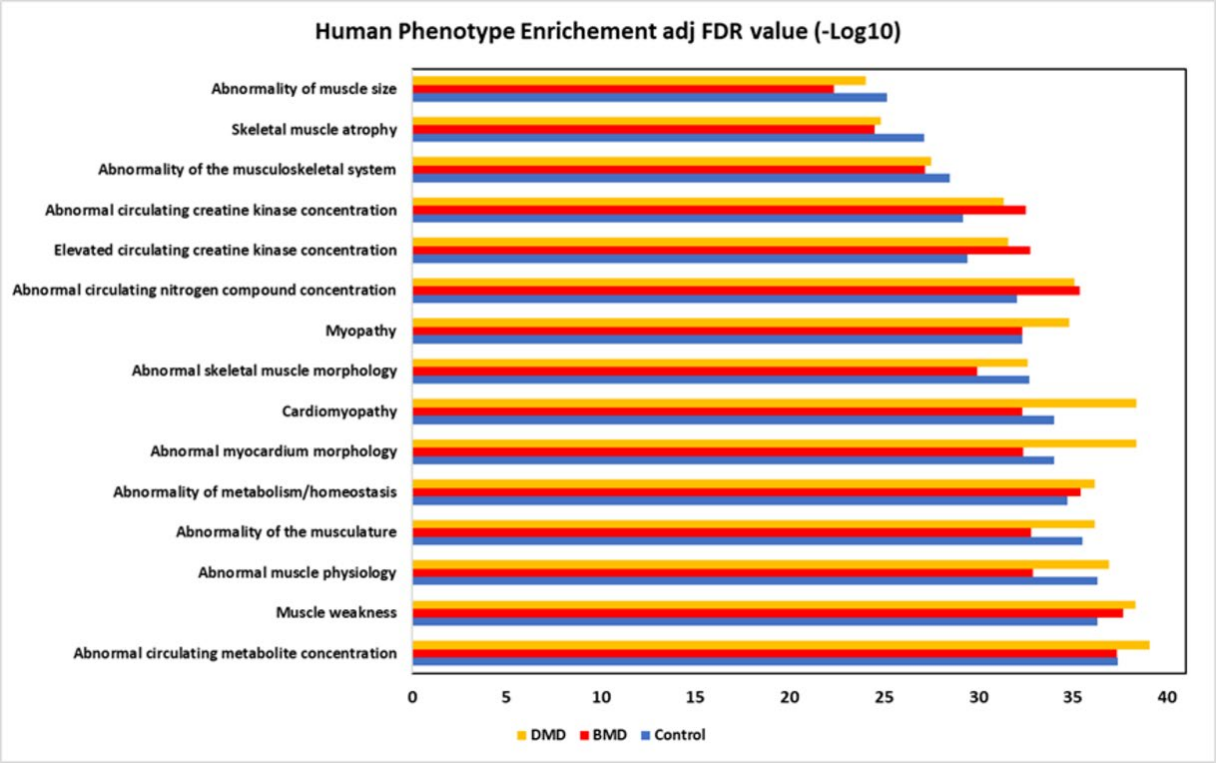
Monarch Initiative classification showing the human phenotype enrichment for the top fifteen human phenotypes based on the adjusted (adj) FDR value.

We found that the three most enriched human phenotypes in the experimental groups were associated with abnormal circulating metabolite concentration (HP:0032180), muscle weakness (HP:0001324) and abnormal muscle physiology (HP:0011804) (Fig. 3; Supplemental Table 7). We observed 161 proteins in abnormal circulating metabolite concentration CG, 144 proteins for DMD, and 154 proteins forBMD (Supplemental Table 8). For abnormal muscle physiology, 238 proteins were found in CG, 203 proteins in DMD, and 222 proteins inBMD (Supplemental Table 8). In the classification of muscle weakness, 146 proteins were found for CG, 134 proteins in DMD, and 140 proteins inBMD (Supplemental Table 8).

#### Signalling pathway analysis showed a decrease in metabolism of proteins

**Fig. 4:**
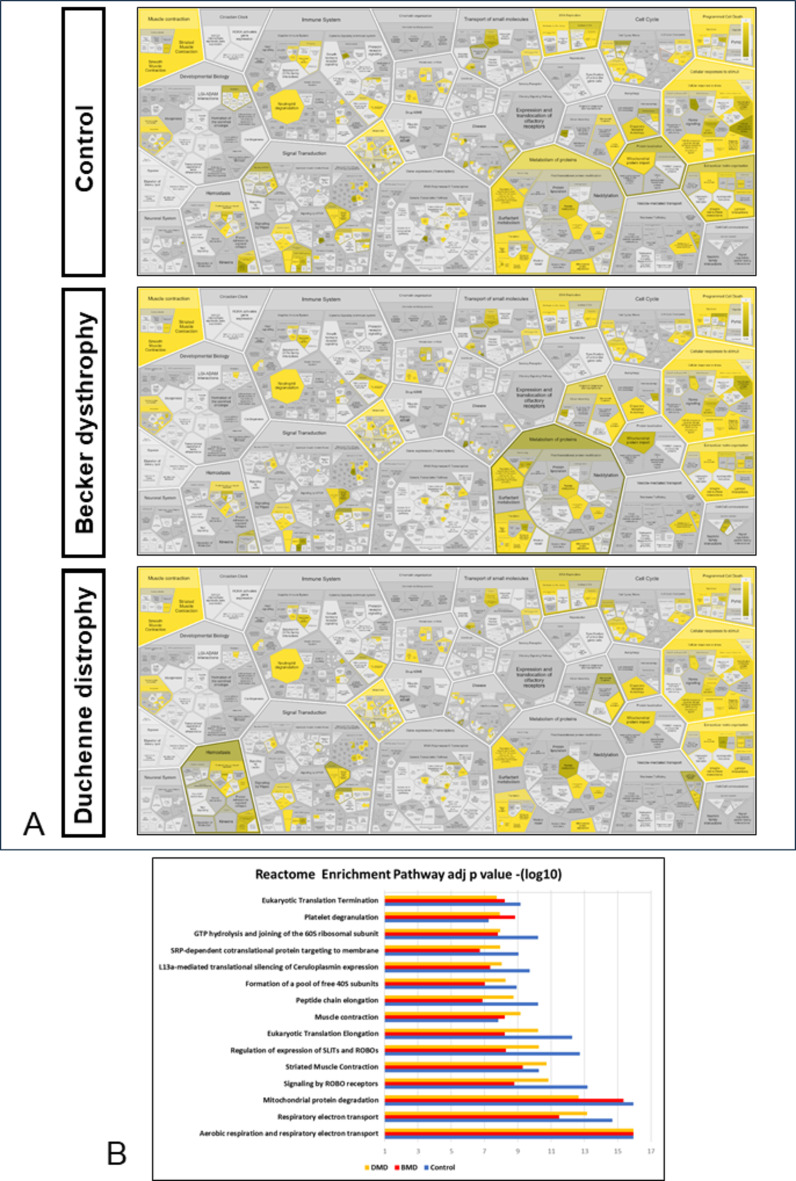
Reactome Pathway Database analysis with all the proteins identified and quantified in each group (CG, BG and DG). (**A**) Reacfoam showing p value coverage for all proteins identified and quantified in control, Becker and Duchenne groups. (**B**) Graphical representation of the top fifteen reactome pathways based on adjusted p value enrichment.

To analyse the alteration of signalling pathways between CG, BG and DG, we performed a Reactome analysis with all proteins identified in each group. The results showed an overall decrease in protein metabolism from CG to BG and DG, respectively (Fig. 4A).Specially processes involved in post translational protein modification as protein methylation and protein deubiquitination as Ub specific processing proteases that were less enriched in BG and DG compared to CG.

Enrichment analysis of the main pathways showed that most of them decreased compared to the control and that mitochondrial protein degradation decreased as dystrophy worsened (Fig. 4B).In the CG a total of 41 proteins were identified, in the BG 38 proteins were identified and in the DG 33 proteins were identified in the mitochondrial protein degradation pathway. Analyzing these proteins we found that 3 proteins were identified only in the CG (ADP/ATP translocase 2, NADH-ubiquinone oxidoreductase chain 5, NADH-ubiquinone oxidoreductase chain 1), and 5 proteins were communs to CG and BG (Fumarate hydratase, mitochondrial, Transcription factor A, mitochondrial, ATP synthase-coupling factor 6 mitochondrial, Short/branched chain specific acyl-CoA dehydrogenase mitochondrial, NADH dehydrogenase [ubiquinone] 1 alpha subcomplex subunit 2) and these 8 proteins were not identified in DG.

#### Becker and Duchenne muscular dystrophy quantitatively alter the molecular signature of muscle tissues

Although the dystrophin abundance alteration in Becker and its severe absence in Duchene has been well established, the possibility to identify and quantify more proteins differentially expressed in these dystrophies could be important to better understand the molecular mechanism involved in these diseases.

Our mass spectrometry analysis revealed that the mean protein abundances of the dystrophin, sarcoglycans and dystroglycan were decreased in BG and DG in comparison to CG (Supplemental Table 9, Supplemental Fig. 2). Although dystrophin was identified and quantified in all groups, including the Duchene group, its mean abundance in Becker and Duchenne was less than 6% compared to the control group which is in accordance with the dystrophin severe absence established for duchenne dystrophy ranging from 0.7 to 7%^30^.

**Fig. 5:**
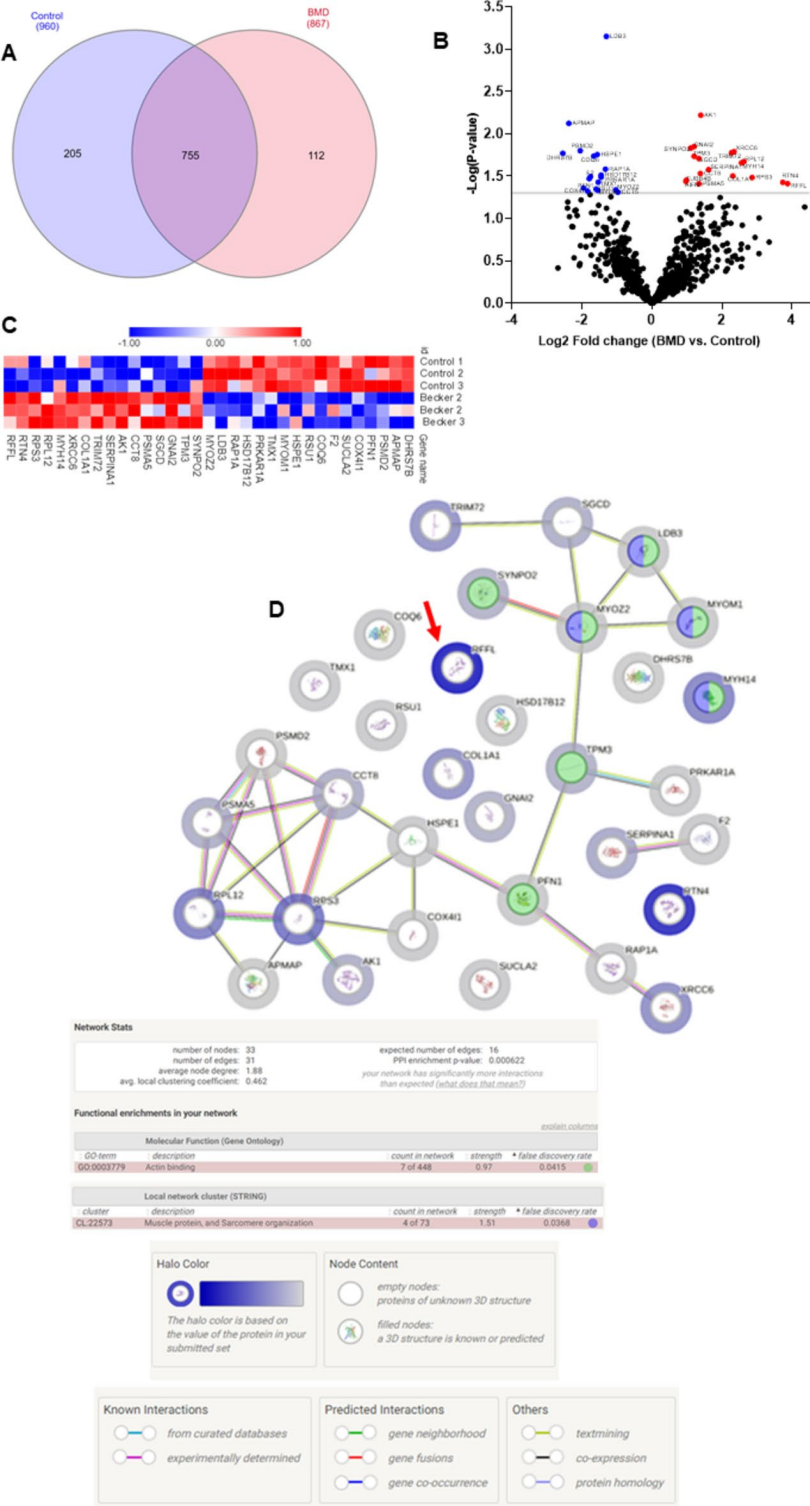
(**A**) Interact Venn Diagram distribution between the 960 proteins identified and quantified in CG and the 867 proteins identified and quantified in BG by our label free proteomic MS/MS data. (**B**) Volcano plots of all proteins from CG and BG. Proteins with increased fold change ratio are indicated by red circles whereas the blue circles denote those proteins presenting a decreased fold change ratio. (**C**) Heat Map of the proteins with a relative fold change higher than 2 (0.5 < FC > 2) for the ratio BG/CG. (**D**) String protein-protein interaction network of the proteins with a relative fold-change higherthan 2 (0.5 < FC > 2) for the ratio BG/CG. The halo colour is based on the FC of the protein.

Comparing the protein distribution between BG and CG (Fig. 5A), we could observe that 755 proteins were common to BG and CG, 112 proteins were identified only in BG and 205 proteins were identified only in CG. The 755 common proteins showed 17 proteins whose differences in abundances were statistically significantly decreased when compared to the control group (Fig. 5B, C; Table 1).

**Table 1:** Proteins with abundance statistically significantly decreased in BG compared to CG.

Uniprot ID	Gene Name	Protein name	Function	*P* value	FC
A0A0C4DGQ8	DHRS7B	Dehydrogenase/reductase SDR family member 7B	Oxidoreductase activity	0.017	0.17
H0Y512	APMAP	Adipocyte plasma membrane-associated protein	Biosynthetic process	0.0075	0.19
Q13200	PSMD2	26 S proteasome non-ATPase regulatory subunit 2	Protein binding	0.0159	0.24
P07737	PFN1	Profilin-1	Actin binding	0.0438	0.26
P13073	COX4I1	Cytochrome c oxidase subunit 4 isoform 1 mitochondrial	Cytochrome c oxidase activity	0.0474	0.28
A0A2R8Y6Y7	SUCLA2	Succinate–CoA ligase [ADP-forming] subunit beta, mitochondrial	ATP-specific succinyl-CoA synthetase	0.0339	0.29
P00734	F2	Prothrombin	Serine-type endopeptidase activity	0.0322	0.29
Q9Y2Z9	COQ6	Ubiquinone biosynthesis monooxygenase COQ6, mitochondrial	Ubiquinone biosynthetic process	0.0183	0.32
Q15404	RSU1	Ras suppressor protein 1	Positive regulation of cell-substrate adhesion	0.0449	0.33
B8ZZL8	HSPE1	10 kDa heat shock protein, mitochondrial	Protein folding chaperone	0.0176	0.34
P52179-2	MYOM1	Isoform 2 of myomesin-1	Component of the vertebrate myofibrillar M band	0.0465	0.34
Q9H3N1	TMX1	Thioredoxin-related transmembrane protein 1	Protein disulfide isomerase activity	0.0373	0.34
P10644	PRKAR1A	cAMP-dependent protein kinase type I-alpha regulatory subunit	cAMP signaling	0.0326	0.36
Q53GQ0	HSD17B12	Very-long-chain 3-oxoacyl-CoA reductase	Lipid metabolism	0.0308	0.36
P62834	RAP1A	Ras-related protein Rap-1 A	GTPase activity	0.0261	0.40
A0A8I5KV04	LDB3	LIM domain-binding protein 3	Metal ion binding	0.0007	0.40
Q9NPC6	MYOZ2	Myozenin-2	Actin binding	0.0460	0.49

On the other hand, we observed 16 proteins whose differences in abundances were statistically significantly increased in BG when compared to CG (Fig. 5B, C; Table 2).

**Table 2:** Proteins with abundance statistically significantly increased in BG compared to CG.

Uniprot ID	Gene name	Protein name	Molecular function	*P* value	FC
Q9UMS6	SYNPO2	Synaptopodin-2	Actin binding	0.0148	2.16
P06753-5	TPM3	Isoform 5 of Tropomyosin alpha-3 chain	Actin binding	0.0184	2.30
P04899	GNAI2	Guanine nucleotide-binding protein G(i) subunit alpha-2	GTP binding	0.0142	2.30
Q92629	SGCD	Delta-sarcoglycan	Component of the sarcoglycan complex	0.0195	2.53
P28066	PSMA5	Proteasome subunit alpha type-5	Protein binding	0.0390	2.54
P50990	CCT8	T-complex protein 1 subunit theta	ATP binding	0.0295	2.61
P00568	AK1	Adenylate kinase isoenzyme 1	Adenylate kinase activity	0.0060	2.63
P01009	SERPINA1	Alpha-1-antitrypsin	Serine protease inhibitor	0.0265	3.07
Q6ZMU5	TRIM72	Tripartite motif-containing protein 72	Phosphatidylserine binding	0.0167	4.83
P02452	COL1A1	Collagen alpha-1(I) chain	Extracellular matrix structural constituent	0.0314	4.96
P12956	XRCC6	X-ray repair cross-complementing protein 6	DNA binding	0.0163	5.08
Q7Z406	MYH14	Myosin-14	Microfilament motor activity	0.0220	5.90
P30050	RPL12	60 S ribosomal protein L12	RNA binding	0.0213	6.19
P23396	RPS3	40 S ribosomal protein S3	DNA N-glycosylase activity	0.0328	7.29
Q9NQC3-3	RTN4	Isoform C of Reticulon-4	RNA binding	0.0374	13.39
Q8WZ73	RFFL	E3 ubiquitin-protein ligase rififylin	Ubiquitin protein ligase binding	0.0388	14.72

To analyse the PPI between these proteins with abundance differences statistically significant we used the STRING platform (Fig. 5D). We found a PPI enrichment p value of 0.000622 for protein interactions within proteins classified in actin binding, muscle protein and sarcomere organization (Fig. 5D). The halo colour in the protein network represents the abundance of the proteins, we could observe the high abundant protein with the strong halo colour (rififylin) and the low abundance proteins with the weak halo colour.

**Fig. 6:**
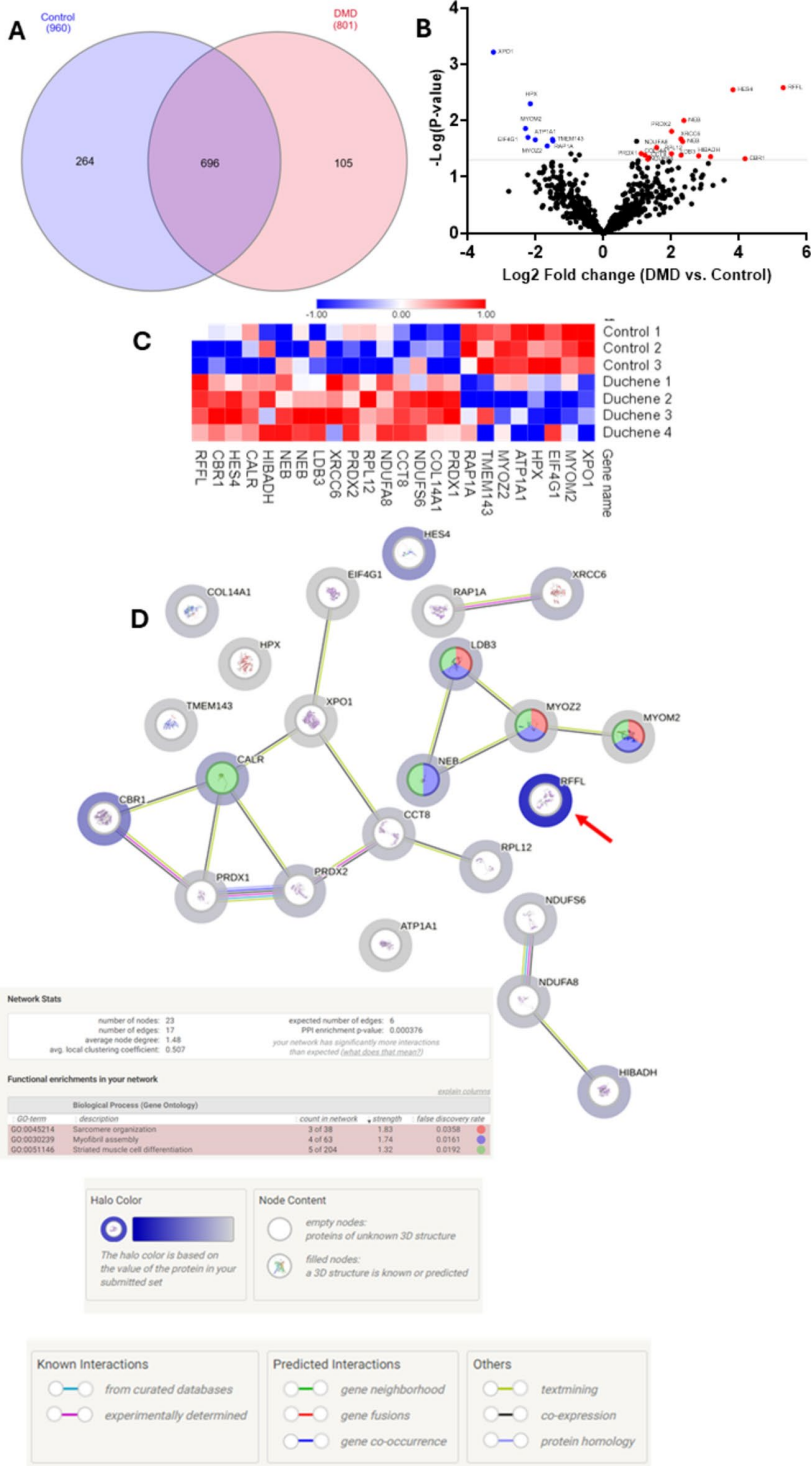
(**A**) Interact Venn Diagram distribution between the 960 proteins identified and quantified in CG and the 801 proteins identified and quantified in DG by our label free proteomic MS/MS data. (**B**) Volcano plots of all proteins from CG and BG. Proteins with increased fold change ratio are indicated by red circles whereas the blue circles denote those proteins presenting a decreased fold change ratio. (**C**) Heat Map of the proteins with a relative fold change higher than 2 (0.5 < FC > 2) for the ratio DG/CG. (**D**) String protein-protein interaction network of the proteins with a relative fold change higher than 2 (0.5 < FC > 2) for the ratio DG/CG. The halo colour is based on the FC of the protein.

Comparing all the identified and quantified proteins between DG and CG (Fig. 6A), we found 696 proteins common to DG and CG, 105 proteins were identified only in DG and 264 proteins were identified only in CG. We observed 8 proteins whose differences in abundances between DG compared to CG were statisticallysignificantlydecreased (Fig. 6B, C; Table 3).

**Table 3:** Proteins with abundance statistically significantly decreased in DG compared to CG.

Uniprot ID	Gene name	Protein name	Molecular function	*P* value	FC
A0A7I2V2S3	XPO1	Exportin-1	Protein transport	0.0006	0.10
P54296	MYOM2	Myomesin-2	Structural constituent of muscle	0.0139	0.20
E7EX73	EIF4G1	Eukaryotic translation initiation factor 4 gamma 1	Translation initiation factor activity	0.0198	0.21
P02790	HPX	Hemopexin	Metal ion binding	0.0050	0.22
P05023	ATP1A1	Sodium/potassium-transporting ATPase subunit alpha-1	P-type sodium: potassium-exchanging transporter activity	0.0219	0.24
Q9NPC6	MYOZ2	Myozenin-2	Actin binding	0.0281	0.31
Q96AN5	TMEM143	Transmembrane protein 143	Protein binding	0.0216	0.35
P62834	RAP1A	Ras-related protein Rap-1 A	GTPase activity	0.0228	0.35

Conversely, we observed 16 proteins whose differences in abundance was statistically significantly increased (Fig. 6B, C; Table 4).

**Table 4:** Proteins with abundance statistically significantly increased in DG compared to CG.

Uniprot ID	Gene name	Protein name	Molecular function	*P* value	FC
Q06830	PRDX1	Peroxiredoxin-1	Peroxidase activity	0.0385	2.16
Q05707	COL14A1	Collagen alpha-1(XIV) chain	Extracellular matrix structural constituent	0.0410	2.33
O75380	NDUFS6	NADH dehydrogenase [ubiquinone] iron-sulfur protein 6, mitochondrial	NADH dehydrogenase (ubiquinone) activity	0.0485	2.48
P50990	CCT8	T-complex protein 1 subunit theta	Protein folding chaperone	0.0453	2.53
P51970	NDUFA8	NADH dehydrogenase [ubiquinone] 1 alpha subcomplex subunit 8	NADH dehydrogenase (ubiquinone) activity	0.0300	2.96
P30050	RPL12	60 S ribosomal protein L12	RNA binding	0.0388	4.01
P32119	PRDX2	Peroxiredoxin-2	Peroxidase activity	0.0155	4.02
P12956	XRCC6	X-ray repair cross-complementing protein 6	DNA binding	0.0212	4.87
O75112-6	LDB3	Isoform 6 of LIM domain-binding protein 3	Cytoskeletal protein binding	0.0412	4.88
P20929-3	NEB	Isoform 3 of Nebulin	Actin binding	0.0235	5.06
P20929-2	NEB	Isoform 2 of Nebulin	Actin binding	0.0100	5.17
P31937	HIBADH	3-Hydroxyisobutyrate dehydrogenase, mitochondrial	3-Hydroxyisobutyrate dehydrogenase activity	0.0422	6.99
A0A7P0T861	CALR	Calreticulin	Calcium ion binding	0.0436	8.92
D6REB3	HES4	Transcription factor HES-4	DNA binding	0.0028	14.12
P16152	CBR1	Carbonyl reductase [NADPH] 1	Carbonyl reductase (NADPH) activity	0.0474	18.06
Q8WZ73	RFFL	E3 ubiquitin-protein ligase rififylin	Ubiquitin protein ligase binding	0.0026	39.44

Analysing the PPI between the proteins with abundance differences statistically significant in the STRING platform we found a PPI enrichment p value of 0.000376 forprotein interactions within proteins classified in sarcomere organization, myofibril assembly and striated muscle cell differentiation. The halo colour in the protein network represents the abundance of the proteins, we could observe the high abundant protein with the strong halo colour (rififylin) and the low abundance proteins with the weak halo colour (Fig. 6D).

**Fig. 7:**
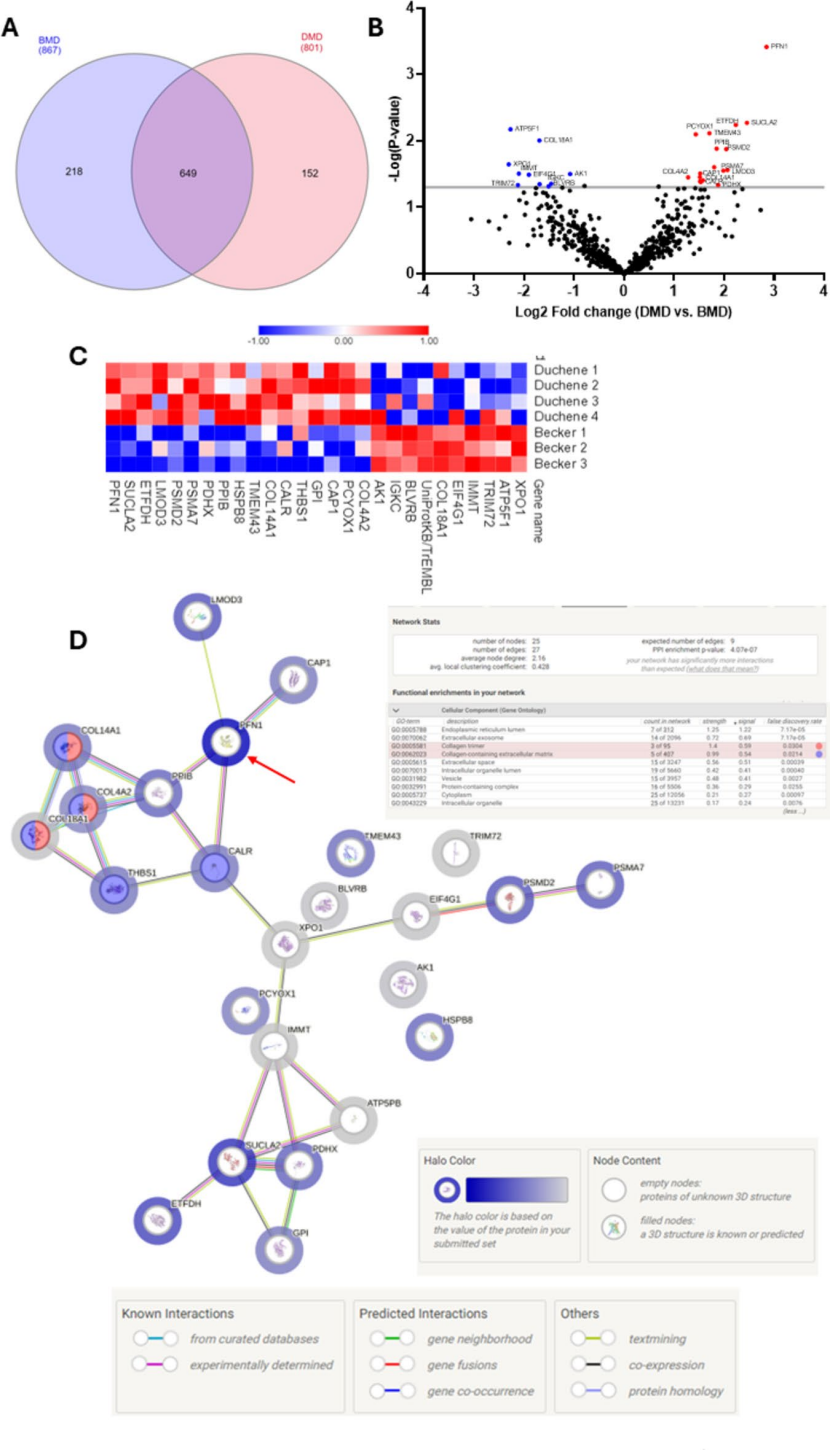
(**A**) Interact Venn Diagram distribution between the 867 proteins identified and quantified in BG and the 801 proteins identified and quantified in DG by our label free proteomic MS/MS data. (**B**) Volcano plots of all proteins from DG and BG. Proteins with increased fold change ratio are indicated by red circles whereas the blue circles denote those proteins presenting a decreased fold change ratio. (**C**) Heat Map of the proteins with a relative fold change higher than 2 (0.5 < FC > 2) for the ratio DG/BG. (**D**) String protein-protein interaction network of the proteins with a relative fold change higher than 2 (0.5 < FC > 2) for the ratio DG/BG. The halo colour is based on the FC of the protein.

Finally, we compared all the identified and quantified protein distribution between DG and BG (Fig. 7A), we could observe that 649 proteins were common to DG and BG, 152 proteins were identified only in DG and 218 proteins were identified only in BG. When comparing the DMD and BMD groups, we observed 10 proteins whose differences in abundance were statistically significantly decreased in DMD compared to BMD (Table 5).

**Table 5:** Proteins with abundance statistically significantly decreased in DG compared to BG.

Uniprot ID	Gene name	Protein name	Molecular function	*P* value	FC
A0A7I2V2S3	XPO1	Exportin-1	Protein transport	0.0226	0.20
P24539	ATP5F1	ATP synthase F(0) complex subunit B1, mitochondrial	Proton-transporting ATP synthase activity	0.0067	0.21
Q6ZMU5	TRIM72	Tripartite motif-containing protein 72	Phosphatidylserine binding	0.0465	0.23
Q16891-2	IMMT	Isoform 2 of MIC complex subunit MIC60	Protein binding	0.0312	0.23
E7EX73	EIF4G1	Eukaryotic translation initiation factor 4 gamma 1	Translation initiation factor activity	0.0325	0.27
P39060	COL18A1	Collagen alpha-1(XVIII) chain	Extracellular matrix structural constituent	0.0450	0.31
P30043	BLVRB	Flavin reductase (NADPH)	Oxidoreductase activity	0.0486	0.35
P01834	IGKC	Immunoglobulin kappa constant	Antigen binding	0.0445	0.37
P00568	AK1	Adenylate kinase isoenzyme 1	Adenylate kinase activity	0.0317	0.47

In contrast, we observed 17 proteins whose differences in abundance were statistically significantly increased when comparing DG vs. BG (Fig. 7B, C; Table 6).

**Table 6:** Proteins with abundance statistically significantly increased in DG compared to BG.

Uniprot ID	Gene name	Protein name	Molecular function	*P* value	FC
P08572	COL4A2	Collagen alpha-2(IV) chain	Extracellular matrix structural constituent	0.0356	2.43
Q9UHG3	PCYOX1	Prenylcysteine oxidase 1	Prenylcysteine oxidase activity	0.0080	2.70
Q01518	CAP1	Adenylyl cyclase-associated protein 1	Actin binding	0.0354	2.86
P06744	GPI	Glucose-6-phosphate isomerase	Glucose-6-phosphate isomerase activity	0.0409	2.86
P07996	THBS1	Thrombospondin-1	Fibronectin binding	0.0311	2.87
A0A7P0T861	CALR	Calreticulin	Calcium ion binding	0.0417	2.89
Q05707	COL14A1	Collagen alpha-1(XIV) chain	Extracellular matrix structural constituent	0.0394	2.98
Q9BTV4	TMEM43	Transmembrane protein 43	Protein binding	0.0077	3.26
A0A6Q8PGM6	HSPB8	Heat shock protein beta-8	Protein homodimerization activity	0.0251	3.48
A0A7P0Z497	PPIB	Peptidyl-prolyl cis-trans isomerase	Peptidyl-prolyl cis-trans isomerase activity	0.0132	3.60
O00330	PDHX	Pyruvate dehydrogenase protein X component, mitochondrial	Acyltransferase activity	0.0463	3.68
O14818	PSMA7	Proteasome subunit alpha type-7	Protein binding	0.0282	3.95
Q13200	PSMD2	26 S proteasome non-ATPase regulatory subunit 2	Protein binding	0.0133	4.12
Q0VAK6	LMOD3	Leiomodin-3	Actin monomer binding	0.0275	4.18
B4DEQ0	ETFDH	Electron transfer flavoprotein-ubiquinone oxidoreductase	Electron-transferring-flavoprotein dehydrogenase activity	0.0058	4.70
A0A2R8Y6Y7	SUCLA2	Succinate–CoA ligase [ADP-forming] subunit beta, mitochondrial	Succinate-CoA ligase (ADP-forming) activity	0.0054	5.48
P07737	PFN1	Profilin-1	Actin binding	0.0004	7.19

The PPI between the proteins with abundance differences statistically significant in the STRING platform showed a PPI enrichment p value of 4.07 × 10^7^ forprotein interactions within proteins classified in collagen trimer and collagen-containing extracellular matrix. The halo colour in the protein network represents the abundance of the proteins, we could observe the high abundant protein with the strong halo colour (Profilin-1) and the low abundance proteins with the weak halo colour (Fig. 7D).

Of the proteins mentioned above, we highlight 2 proteins with decreased abundance in both DMD and BMD, when compared to control: Myozenin-2 (MYOZ2; Q9NPC6; fold change: 0.49) and Myomesin-2 (MYOM2; P54296). Four proteins were significantly increased to both dystrophies compared to control: T-complex protein 1 subunit theta (CCT8; P50990); X-ray repair cross-complementing protein 6 (XRCC6; P12956); 60 S ribosomal protein L12 (RPL12; P30050) and E3 ubiquitin-protein ligase rififylin (RFFL; Q8WZ73). We also observed 2 proteins upregulated in DMD compared with both CG and BMD: Calreticulin (A0A7P0T861), Collagen alpha-1(XIV) chain (COL14A1; Q05707). And finally, we observed 2 other proteins whose abundances were decreased in DMD compared with both CG and BMD: Exportin-1 (A0A7I2V2S3) and Eukaryotic translation initiation factor 4 gamma 1 (EIF4G1; E7EX73).

We also find one protein whose abundance was increased in BG, usually associated to Limb Girdle Muscular Dystrophy (LGMD), Delta-sarcoglycan (SGCD; Q92629) and two other proteins whose abundance was decreased, associated to muscular disorders in Congenital Myopathy: Tropomyosin alpha-3 chain (TPM3; P06753-5) and Leiomodin-3 (LMOD3; Q0VAK6).

## Discussion

Although Duchenne and Becker Muscular Dystrophies are directly related to the dystrophin protein, it is very important to find other proteins that contribute to the clinical heterogeneity among patients with this disease. Two proteins stand out for being less abundant in both types of dystrophies compared to the control, suggesting the loss or silencing of these proteins during the degenerative process caused by the dystrophy. One of them, Myozenin-2, is a Z-disc protein exclusively observed in striated muscles, which is strongly associated with studies of human hypertrophic cardiomyopathy^31^. Ruggiero et al. concluded that alterations in the Z disc may favour the induction of cardiac hypertrophy caused by mutations in the MYOZ2 gene^32^. Murphy et al. found a reduced abundance of the isoform 1 of myozenin comparing the dystrophic mdx-4cv mouse model versus age-matched leg muscle microsomes during the investigation of the differential protein expression patterns between dystrophic and normal skeletal muscle^33^.

The other protein, myomesin-2, with reduced abundance in both BMD and DMD is a M-line sarcomere protein important in the monitoring of sarcomere assembly and integrity as it is the last protein to be incorporated into the sarcomere. Myomesin assays have been described as a more specific method for detecting sarcomere damage than muscle creatine kinase in striated muscle disease^34^.

Our Gene Ontology analyses using STRING emphasize the function of this protein, since it was detected in Molecular Functions related to the structural activity of the cell. The importance of proteins from sarcomere in maintaining the integrity of skeletal striated muscle cells is well understood, which raises the hypothesis of a probable loss of these proteins in dystrophic tissue.

One protein was highlighted, the protein E3 ubiquitin-protein ligase rififylin, that had the highest increased in both BMD (fold change 14.72) and in DMD (fold change 39.44). This protein has been described as a risk factor for cardiac repolarization via ubiquitination of multiple proteins that differently affect different potassium channels and cardiac action potential duration^35,36^. Also known as an apoptosis inhibitor, rififylin protein regulates several biological processes via ubiquitination leading to proteasomal degradation of several target proteins as caspase-8 and caspase-10 as well as p53^37,38^.

Of the proteins found with abundance decreased only in DMD, suggesting a more aggressive loss in this type of dystrophy compared to its milder variant BMD and the control group, Exportin-1 plays an essential role in the nucleocytoplasmic trafficking of key transcription factor proteins^39^. Suárez-Sánchez et al. showed that Dp71d, a shortened form of dystrophin in Duchene muscular dystrophy, is transported between the nucleus and cytoplasm by the conventional nuclear transporters, importin (IMP) α/β and the exportin-1 CRM1^40^. Exportin-1 have been described to interact with polyglutamine (polyQ) proteins that are produced in patients with amyotrophic lateral sclerosis (ALS) and frontotemporal dementia (FTD). In these patients the defective nuclear export activity regulated by exportin-1 was observed^41,42^. Some selective inhibitors of nuclear export (SINEs) have been developed initially for cancer therapies, however, recent discoveries have demonstrated that exportin-1 could serve as a target in neurological and neuromuscular disorders containing pathogenic expansion repeats affecting export in protein activity^39,43^.

The Eukaryotic translation initiation factor 4 gamma 1 (EIF4G1) has been related to breast cancer and Parkinson disease^44,45^. Sanson et al. described the involvement of EIF4G2 in the mitochondrial metabolism of glucocorticoid used in Duchenne patients’ treatment, through its target by miR379^46^.

Of the proteins found increased only in DMD, which suggest an overexpression in this type of dystrophy in relation to CG and BMD, Calreticulin is a Ca2 + binding protein in the endoplasmic reticulum of muscle and nonmuscle cells^47^. Morales, et al., 2024 found an increase of calreticulin at a transcriptional level in a canine DMD model. The authors concluded that increase in the cytosolic calcium levels were an important mechanism in the regulation of muscle degradation in DMD. High levels of Ca2 + that also accumulates in mitochondria contributes to mitochondrial dysfunction and muscle damage in DMD^48–50^.

The protein Collagen alpha-1(XIV) chain plays a role in integrating collagen fibrils^51^. It is known that patients with DMD express higher levels of collagen and extracellular matrix factors^52,53^. Capitanio et al. using an in gel label free proteomic strategy found an increase in extracellular matrix proteins specially collagens in DMD compared to BMD^18^. In our proteomic analysis we also found collagen classified as contained in extracellular matrix increased in DMD compared to BMD patients. Comparing DMD and BMD, the profilin-1 protein was highlighted with a FC of 7.19 times higher in DMD compared to BMD. Profilin-1 is a protein that binds to actin and affects the organization of the cytoskeleton, however, in a higher concentration it is capable of preventing actin polymerization^54,55^.

The Delta-sarcoglycan protein (SGCD), which is usually associated with Limb Girdle Muscular Dystrophy (LGMD), was found to be more abundant in BMD compared to control. This transmembrane protein is a member of DGC which binds to dystroglycans, which in turn, were associated with the third domain of Dystrophin. This increase in expression was also reported by Zanotti et al. who demonstrated a significant increase in delta-sarcoglycan in patients with Becker, compared to other types of sarcoglycans^56^. Our findings may contribute with information that may justify reports of under diagnosis of BMD that are sometimes classified as LGMD^22,23^. Our analysis on the Monarch initiative revealed that this protein is associated with the Human Phenotypes revealing participation in muscle weakness, physiology and abnormal concentration of metabolites. We raised the hypothesis of SGCD overexpression as a cellular strategy for correction in the face of damage, although further studies are necessary to evaluate this hypothesis.

Of the proteins that were less abundant in BMDwe highlight Leiomodin-3 (LMOD3), which is a slow-growing end-binding protein of filamentous actin, and Isoform 5 of Tropomyosin alpha-3 chain (TPM3), which is a troponin-actin complex-binding protein both proteins have been associated with congenital myopathies^57,58^. These proteins were classified in our gene ontology analysis using STRING in the most enriched molecular functions and human phenotypes demonstrating their participation have been related to structural activities and abnormal functions of muscle.

The data found in our study demonstrate that we have taken an important step in the investigation of biomarkers and molecular signatures of DMD and BMD. Although the diagnosis of these diseases is currently largely linked to genetic investigation, in inconclusive cases, muscle biopsy is necessary to determine the presence of Dystrophin. It is important to emphasize that diagnosis through genetic tests is not accessible to the entire population, as they are more expensive^59^. Therefore, our studies have contributed to the knowledge of more proteins involved in DMD and BMD pathologies.

## Conclusion

This study shows a LC-MS/MS label free proteomic approach analysis of muscle tissues from patients with Becker and Duchenne muscular dystrophies. Among the proteins identified and quantified, most were associated with structural activities of the muscle cell and physiological abnormalities of muscle or neurodegenerative diseases. Due to the challenges of obtaining more biopsy samples from rare pediatric diseases, new studies are necessary to validate our results. However, our findings were corroborated by the literature, which confirms a clear path in the search for new targets in DMD and BMD diseases.

## Electronic supplementary material

Below is the link to the electronic supplementary material.

Supplementary Material 1

Supplementary Material 2

Supplementary Material 3

Supplementary Material 4

Supplementary Material 5

Supplementary Material 6

Supplementary Material 7

Supplementary Material 8

Supplementary Material 9

Supplementary Material 10

Supplementary Material 11

## Data Availability

Data Availability: The mass spectrometry proteomics data have been deposited to the ProteomeXchange Consortium via the PRIDE partner repository with the dataset identifier PXD050694, at [http://proteomecentral.proteomexchange.org/cgi/GetDataset].
