# Supplementary material for: Label-free proteomic analysis of Duchenne and Becker muscular dystrophy showed decreased sarcomere proteins and increased ubiquitination-related proteins
Source: Sci Rep. 2025 Jan 26;15:3293. doi: 10.1038/s41598-025-87995-5 (PMC11770181; doi:10.1038/s41598-025-87995-5)
Supplement: Supplementary file 2 — Supplementary Material 2 [file 41598_2025_87995_MOESM2_ESM.pdf]

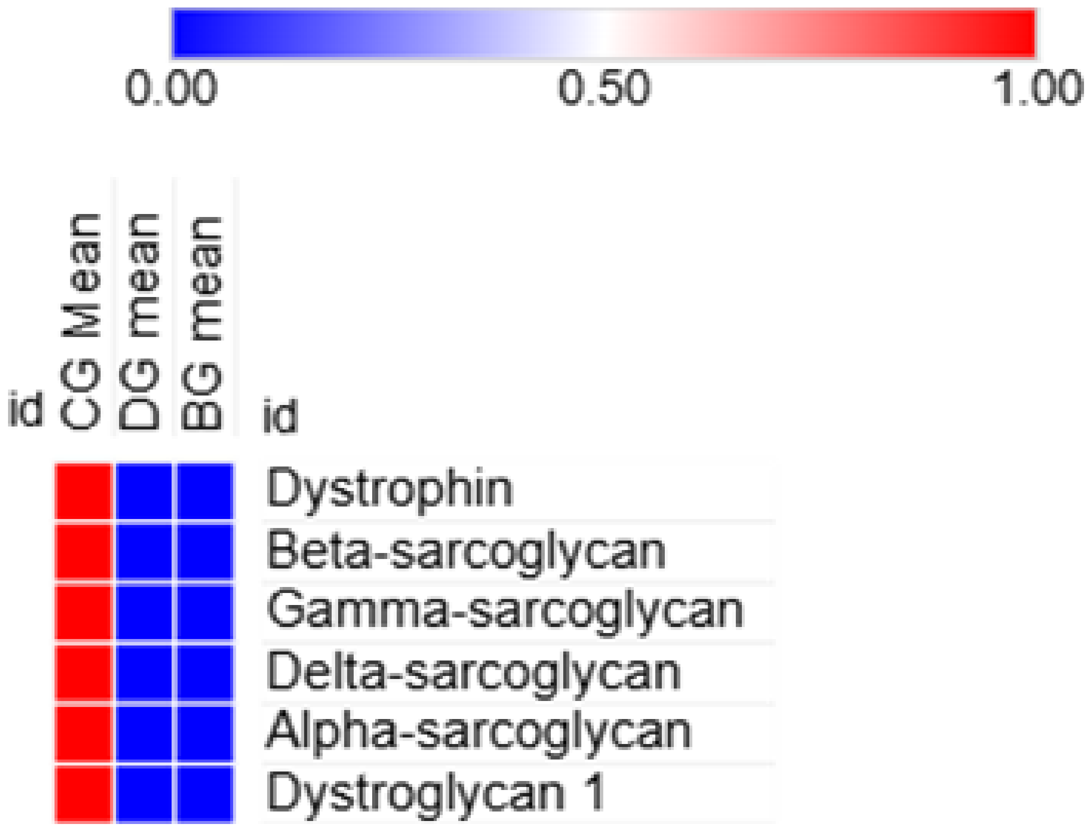

Supplemental figure 2 – Heat map with the dystrophin, sarcoglycan and dystroglycan proteins identified and quantified by our mass spectrometry analysis. The protein abundances of the dystrophin, sarcoglycans and dystroglycan were decreased in DG and BG in comparison to CG.
