# Supplementary material for: Label-free proteomic analysis of Duchenne and Becker muscular dystrophy showed decreased sarcomere proteins and increased ubiquitination-related proteins
Source: Sci Rep. 2025 Jan 26;15:3293. doi: 10.1038/s41598-025-87995-5 (PMC11770181; doi:10.1038/s41598-025-87995-5)
Supplement: Supplementary file 4 — Supplementary Material 4 [file 41598_2025_87995_MOESM4_ESM.pdf]

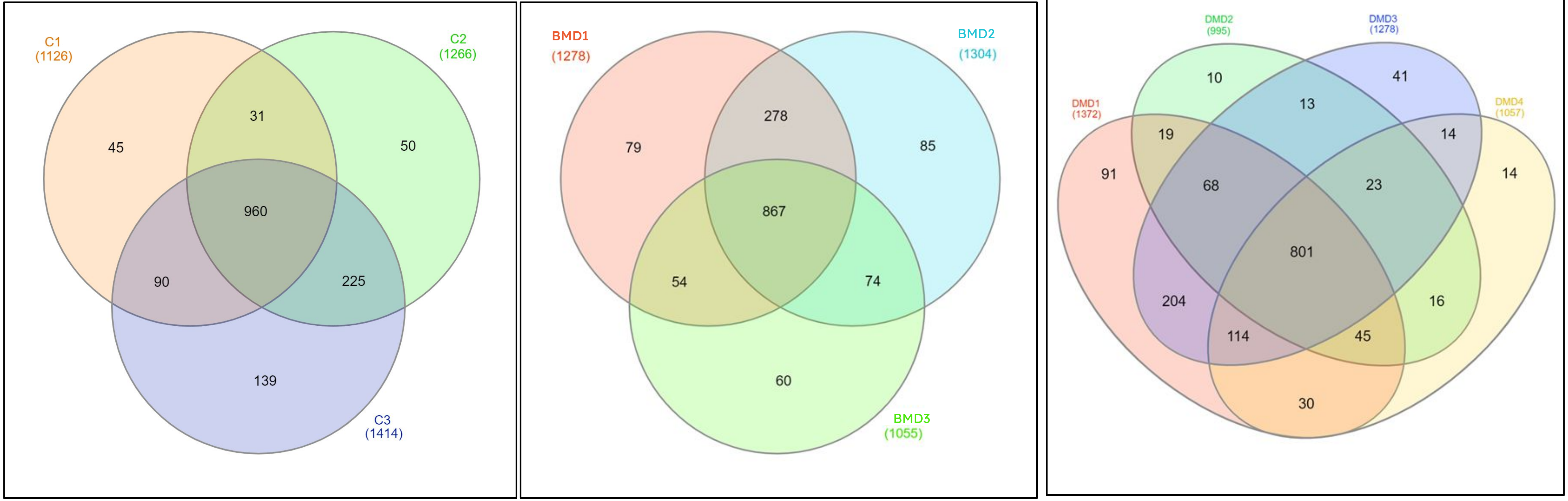

Supplemental figure 1 – Venn diagrams showing protein distribution between the 3 control samples, the 3 Becker dystrophy samples and the 4 Duchenne dystrophy samples. (A) A total of 1,126 proteins were identified in control 1 (C1), 1,266 proteins were identified in control 2 (C2) and 1,413 proteins were identified in control 3 (C3). Among these, 960 proteins were common to all 3 controls and were referred as control group (CG) (B) In Becker Muscular Dystrophy 1 (BMD1) 1,278 proteins were identified; in BMD2 (B2) 1,3034 proteins were identified; and in BMD 3 (B3) 1,055 proteins were identified. Among these, 867 proteins were common to all the 3 Becker patient samples and were referred as BMD group (BG)(C) In Duchenne Muscular Dystrophy 1 (D1) 1,372 proteins were identified; in DMD 2 (D2) 995 proteins were identified; in DMD 3 (D3) 1,278 proteins were identified; and in DMD 4 (D4) 1,057 proteins were identified. Among these, 801 proteins were common to all the 4 DMD patients and were referred to as DMD group (DG).
